# Using wasps as a tool to restore a functioning vine grape mycobiota and preserve the mycobial “terroir”

**DOI:** 10.1038/s41598-023-43541-9

**Published:** 2023-10-02

**Authors:** Monica Di Paola, Agnese Gori, Irene Stefanini, Niccolò Meriggi, Sonia Renzi, Stefano Nenciarini, Benedetta Cerasuolo, Marco Moriondo, Riccardo Romoli, Giuseppe Pieraccini, David Baracchi, Francesco Turillazzi, Stefano Turillazzi, Duccio Cavalieri

**Affiliations:** 1 Department of Biology, University of Florence, via Madonna del Piano 6, Sesto Fiorentino, 50019 Florence, Italy; 2 Department of Life Sciences and Systems Biology, University of Turin, Turin, Italy; 3 National Research Council, Bioeconomy Institute, Sesto Fiorentino, 50019 Florence, Italy; 4 Mass Spectrometry Centre (CISM), University of Florence, via U. Schiff, 6, Sesto Fiorentino, 50019 Florence, Italy; 5 LABREMMA-Laboratory for Medical Entomotherapy, Microbiology and Environment, University of Florence, Sesto Fiorentino, 50019 Florence, Italy

**Keywords:** Biodiversity, Microbiome

## Abstract

In the last one-hundred years, the exponential expansion of wine making has artificialized the agricultural landscape as well as its microbial diversity, spreading human selected *Saccharomyces cerevisiae* strains. Evidence showed that social wasps can harbor a significant fraction of the yeast phenotypic diversity of a given area of wine production, allowing different strains to overwinter and mate in their gut. The integrity of the wasp-yeast ecological interaction is of paramount importance to maintain the resilience of microbial populations associated to wine aromatic profiles. In a field experiment, we verified whether *Polistes dominula* wasps, reared in laboratory and fed with a traceable *S. cerevisiae* strain, could be a useful tool to drive the controlled yeast dispersion directly on grapes. The demonstration of the biotechnological potential of social insects in organic wine farming lays the foundations for multiple applications including maintenance of microbial biodiversity and rewilding vineyards through the introduction of wasp associated microbiomes.

## Introduction

In the industry of fermented products, selected yeast strains are used to trigger, control, and standardize the fermentation process and to ensure a product stable over time with the desired flavors and aromatic bouquet. The organoleptic characteristics of both the fermented musts and the resulting wine depend not only on the vine plant cultivar, but also on geographical traits defined by the vineyard “terroir”^1–5^. Despite the term “terroir” initially referring to factors associated with the vineyard pedology and landscape^6,7^, it is nowadays recognized that the microbial populations are key players in the terroir^1,8,9^. Metagenomic studies are highlighting diverse microbial communities on grapes characterized by differences associated to the geography, for fungal more than for bacterial populations^10^ and indicate that must microbial populations originate from the environment neighboring the vineyard^5^.

In this contest, social insects (such as bees, wasps, hornets, and ants) play a fundamental role in spreading microorganisms in the natural environment. Wasps have been shown to maintain *Saccharomyces cerevisiae* in their intestines all year around and contribute to the dispersion of the yeast in the environment^11,12^. Despite wasps having been shown to harbor strains that represent a large fraction of the yeast phenotypic diversity of a given area of wine production^13^, the actual effectiveness of wasps to vector *S. cerevisiae* as well as other microbial populations in the vineyard environment remains unproven. In fact, besides *S. cerevisiae*, other microbial species present in fresh musts may have major impacts on the success of fermentation, by either contributing to the alcoholic fermentation process and in the definition of the organoleptic characteristics of the final product or hindering the process by producing unpleasant flavor and taste^14^.

To verify the vector potential of social wasps, we performed a controlled experiment in the field by introducing *Polistes dominula* social paper wasp fed with a selected *S. cerevisiae* strain in an experimental vineyard. Using genetically selectable *S. cerevisiae* strains in combination with meta-barcoding analyses of the ITS1-4 fungal region, we were able to track the spread of this strain from wasps to the vineyard environment and from the vineyard to the fermented musts.

Our field experiment allowed us to demonstrate to what extent social wasps could be used as biological tools to drive the spreading of desirable yeasts directly into the vineyard to influence the microbiota of grapes and fermenting must and ultimately control the organoleptic characteristics of the final product.

## Results

### Experimental set-up in laboratory and in vineyard

**Figure 1:**
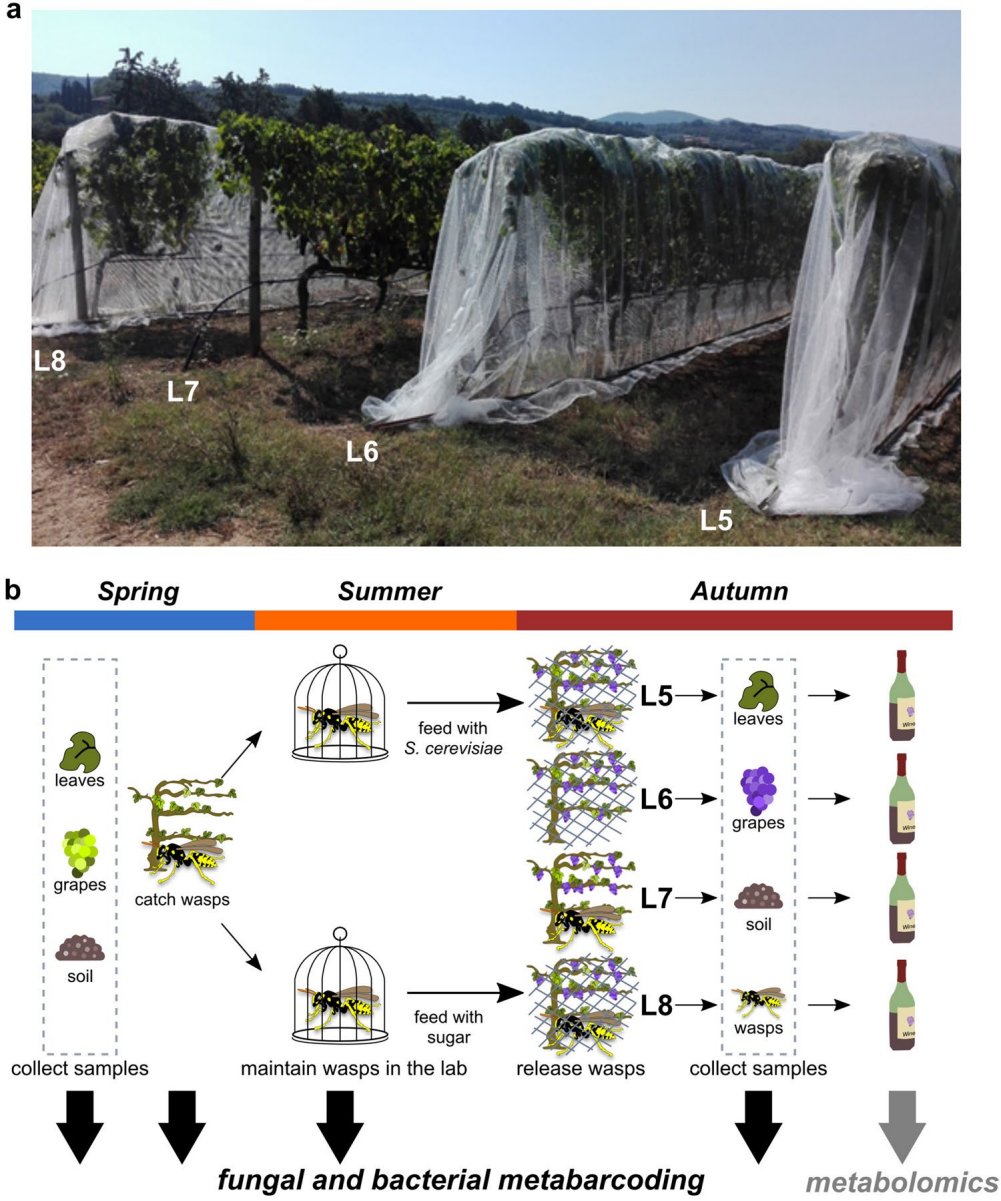
Experiment vineyard and study design. (**a**) Field setting, with experimental vineyard lines (L5, L6, and L8) covered with nets and L7 net-free. (**b**) Scheme of the treatments and samples collection, with details on timings and metabarcoding and metabolomics analyses.

Before carrying out the field experiment (Fig. 1) to assess the ability of social wasps to release and spread yeast cells into the vineyard, we firstly set up an experiment in the laboratory to assess whether *P. dominula* wasps were able to pierce the grape skin of ripe berries as previously demonstrated for *Vespa crabro*^11^. We found that wasps, either alone or in groups, were able to break the grapes, with individual wasps taking longer to pierce the skin (Supplementary Fig. 1; ANOVA p = 0.0261). We did not find significant differences in the times needed by female and male wasps (either alone or in groups), to break the grapes. Thus we decided to perform the experiment with *P. dominula* instead of *V. crabro*, because they are easier to rear in the laboratory and less dangerous when handled.

In the experimental vineyards, the Ca’Marcanda vineyard (Castagneto Carducci, Tuscany, Italy), three plant-rows were individually covered with dense nets, to avoid the escape of the released wasps and to prevent the entrance and exit of other wasps and other Hymenoptera (Fig. 1a). An additional plant-row was maintained free from the net as the control to monitor the natural presence of microorganisms in the vineyard (L7; Fig. 1a).

A group of *P. dominula* fed with sugar and a selected *Saccharomyces cerevisiae* 1014 strain (resistant to 5,5,5-trifluoro-dl-leucine (5,5,5-TFL) and 5-fluoro-orotic acid (5-FOA) (see “Methods”) and a wasp control group fed with sugar and sterile water were prepared in the laboratory for release in the vineyard (Fig. 1b). To determine experimentally if the selected yeast strain could be spread on ripe grapes by wasps used as vectors, the group of wasps fed in the laboratory with *S*. *cerevisiae* cells was released underneath the net in a vineyard plant-line (L5; Fig. 1a). The group of control wasps was released underneath the net in another line (L8; Fig. 1a). To assess the grapes’ microbial population in absence of wasps, a plant line (L6; Fig. 1a) was covered with the net and no wasps were released underneath it.

To ensure that the presence of the nets does not cause stress to the vine plants and does not alter the microclimate of the entire plant row, we monitored the microclimate (temperature and humidity) and plant stress (water potential and leaf heating) of the vineyard plant-rows (Supplementary Fig. 2). The overall picture given by the micrometeorological (temperature, T, and relative humidity, RH), the eco-physiological (Fapar, leaf water potential) and cluster development (sugar content) analyses carried out from spring to summer (at harvest) indicated that the net did not significantly modify the vine growth conditions as compared to the control (Supplementary Information). Differences in T and RH were occasionally found during the day between covered rows, as the likely effect of local conditions. Leaf water potential recorded at harvest (in September) in the upper, central, and lower part of L5 (covered by net) and L7 (uncovered) did not show any significant differences between plant-rows in the morning or at midday (Supplementary Information).

Once the experimental vineyard was prepared, sample collection, metabarcoding and metabolomic analyses were performed as reported in Fig. 1b.

### Tracking S. cerevisiae cells on grapes, wasps and musts

At grape harvest, wasps previously released underneath the net in L5 (plant row with wasps fed with yeast cells) and L8 (wasps not fed with yeast) were recaptured for characterization of their gut fungal microbiome (Fig. 1b). At the same time, ripe grapes (pristine and injured) were collected from each experimental plant-row for mycobiota characterization and for spontaneous fermentation (Fig. 1b).

**Figure 2:**
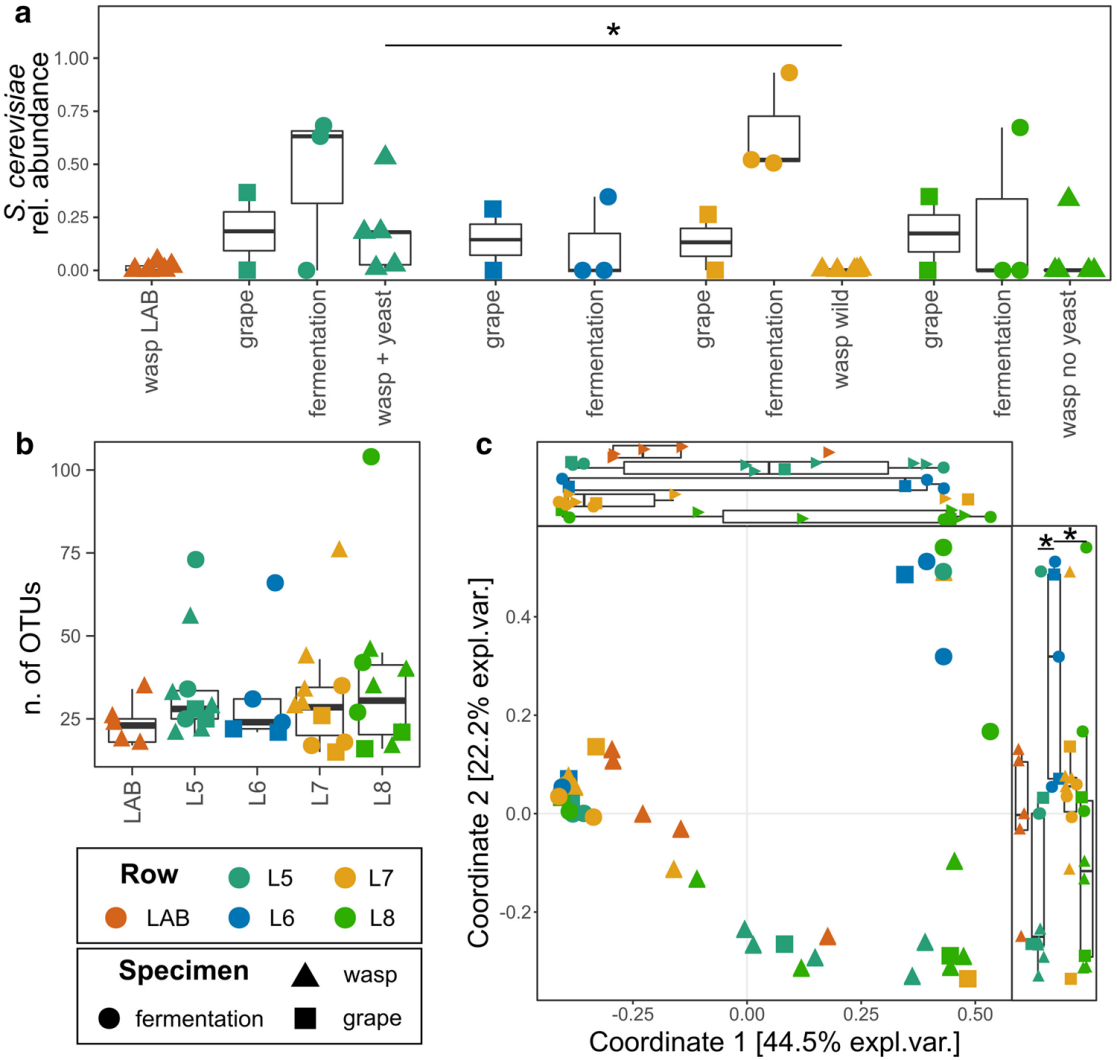
Mycobiota characterization of the experimental vineyard. (**a**) *S. cerevisiae* relative abundance in all samples grouped according to the different environment (laboratory and experimental vine plant rows); (**b**) observed alpha diversity; (**c**) beta diversity—PCoA on weighted UniFrac distances; the boxplots on the right and above the PCoA plot report the distribution of samples, grouped according to the vineyard row, along the first and second coordinate, respectively. *Wilcoxon–Mann–Whitney p-value < 0.05.

We assessed whether wasps fed with *S. cerevisiae* cells showed the expected high relative abundances of OTUs for this fungal species in their guts after the release in the field (Supplementary Table 2a and Fig. 2a).

The comparison confirmed that the relative abundance of *S. cerevisiae* was significantly higher in the guts of wasps fed with the yeast strain and released in L5 than in wasps caught outside the net (referred to as “wasp wild”, captured in L7) (Wilcoxon–Mann–Whitney p-value < 0.05, Fig. 2a). Unsurprisingly, must fermentation samples showed the highest *S. cerevisiae* abundances and the musts obtained from grapes collected from the row covered by the net in the absence of wasps (L6) showed a lower (not significantly different) *S. cerevisiae* abundance compared to the other musts (Fig. 2a). Conversely, the relative abundance of *S. cerevisiae* did not differ among grapes sampled in the analyzed vineyard rows nor among the wine musts obtained from them (Fig. 2a).

By culturing the wasp gut contents and the grapes skin and pulp mycobiota onto selective media including 5,5,5-trifluoro-dl-leucine (5,5,5-TFL) and 5-fluoro-orotic acid (5-FOA) (toward which the 1014 strain fed to wasps is resistant), we could verify that the 1014 yeast strain had been transferred by wasps to the grapes in the corresponding vineyard row (L5). In fact, as expected, 5,5,5-TFL and 5-FOA resistant fungal colonies were absent in samples from the vineyard rows not exposed to the wasps fed with the selected strain (Supplementary Fig. 3), whereas on the grapes exposed to the treated wasps the resistant strains were found up to concentrations of 5.44 × 10^5^ cfu/ml, higher than the one estimated for *S. cerevisiae* cells in fresh wine musts^15^.

### Impact of wasp-vectors release on fungal microbiota diversity of terroir

Upon confirmation of the ability of wasps to act as vectors of the *S. cerevisiae* strain, we evaluated whether the fungal population in grapes and in the resulting musts was modified. Aiming at this, alpha and beta diversities (Fig. 2b,c) of the microbiota of grapes collected in Spring and in Summer (at different maturation states), of musts from grapes of the four different plant rows, and gut of wasps both reared in laboratory and released into the vineyard were compared. Intriguingly, we did not find significant differences among alpha diversities of the fungal microbiota of wasps reared in the laboratory or caught in the vineyard after release nor among grapes harvested from the analyzed vineyard rows (Wilcoxon–Mann–Whitney p > 0.05, Fig. 2b). Conversely, beta diversities highlighted differences in the compositions of the mycobiota of analyzed samples (Supplementary Table 2b,c and Fig. 2c). Bray–Curtis, weighted and unweighted UniFrac distances grouped samples according to the vineyard rows, suggesting that the presence or absence of wasps, per se, either inoculated or not inoculated with yeast, influences the composition of fungal communities in terms of relative fractions of species and of phylogenetic distances among them (permANOVA p.value < 0.05, Supplementary Table 2c). Samples from L6 (plant-row without wasps underneath the net) differed from these of plant-row L5 (with wasps fed with *S. cerevisiae*) and L8 (with control wasps) according to the second coordinate of the PCoAs based on weighted UniFrac (Fig. 2c) and Bray–Curtis (Supplementary Fig. 4) distances. Conversely, the second coordinate of the PCoA based on unweighted UniFrac distances (Supplementary Fig. 4) highlighted significant differences among samples from L5 (with wasp fed with yeast) and L7 (free from the net), among L5 and L6 (without wasp), and among L7 and L8 (with control wasps) samples. In addition, significant differences were observed between the distribution of the mycobiota of wasps reared in the laboratory (“LAB” in Fig. 2c) and samples from the experimental vineyard rows (Supplementary Table 2d and Supplementary Fig. 4).

**Figure 3:**
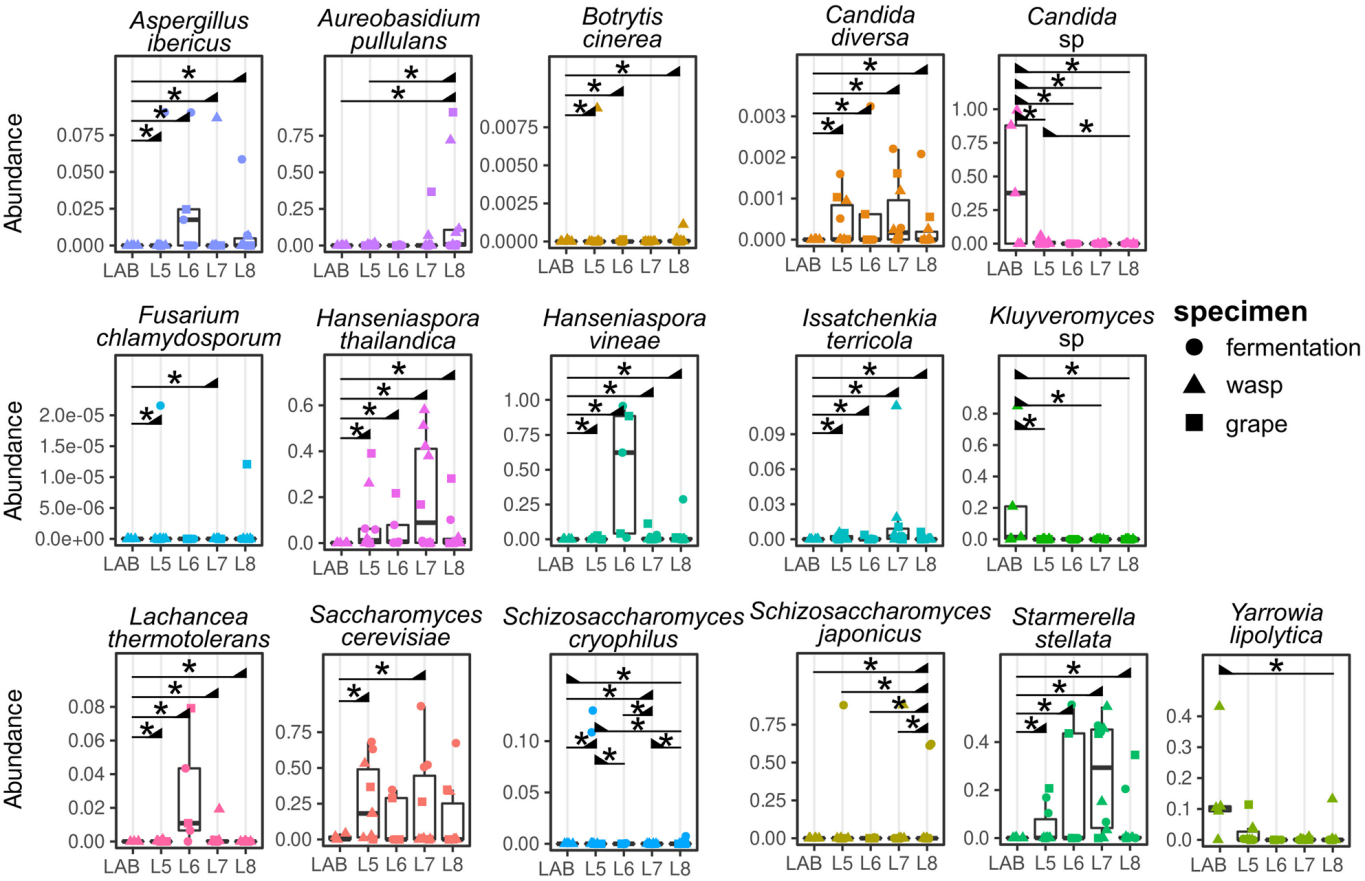
Fungal OTUs with relative abundances significantly differ in the analyzed samples. The relative abundance of each fungal taxon was compared by means of Negative Binomial distribution analysis among samples grouped according to the vineyard of origin or wasps reared in the laboratory. *Wald test p-value corrected by multiple testing < 0.05; the black triangles indicate, among the groups of samples connected by the horizontal line, which group bears the highest abundance of the taxon.

Considering the observed differences among the mycobiota of samples collected in the vineyard rows, we then evaluated whether these differences were ascribable to specific fungal species (Supplementary Tables 2e; 3 and Supplementary Information). Sixteen fungal taxa showed significantly different abundances in samples from different vineyard rows (Supplementary Table 1e and Fig. 3). *Aspergillus ibericus*, *Candida diversa*, *Hanseniaspora thailandica*, *H. vineae*, *Issatchenkia terricola*, *Lachancea thermotolerans*, and *Starmerella stellata* were significantly less abundant in laboratory-reared wasps compared to samples collected from any vineyard row (Fig. 3).

Conversely, *Candida* sp. (OTU not identified at the species level) was significantly more abundant in reared wasps than in any other group of samples. In addition, *Botrytis cinerea* and *Kluyveromyces* sp. were respectively less and more abundant in reared wasps compared to samples from L5, L6, and L8, whereas *Fusarium chlamydosporium* and *S. cerevisiae* were less abundant in reared wasps compared to L5 and L8 samples. *Schizosaccharomyces cryophilus*, a fission yeast, was more abundant in samples from L5 and L7 compared to samples from L8 and to wasps reared in the lab. *Aureobasidium pullulan*s and *Schizosaccharomyces japonicus* were more abundant in L8 samples compared to reared wasps and L5 samples or to every other group of samples, respectively, whereas *Yarrowia lipolytica* was less abundant in L8 compared to reared wasps (Fig. 3).

### Grape’s fermentation and analysis of volatile metabolites

**Figure 4:**
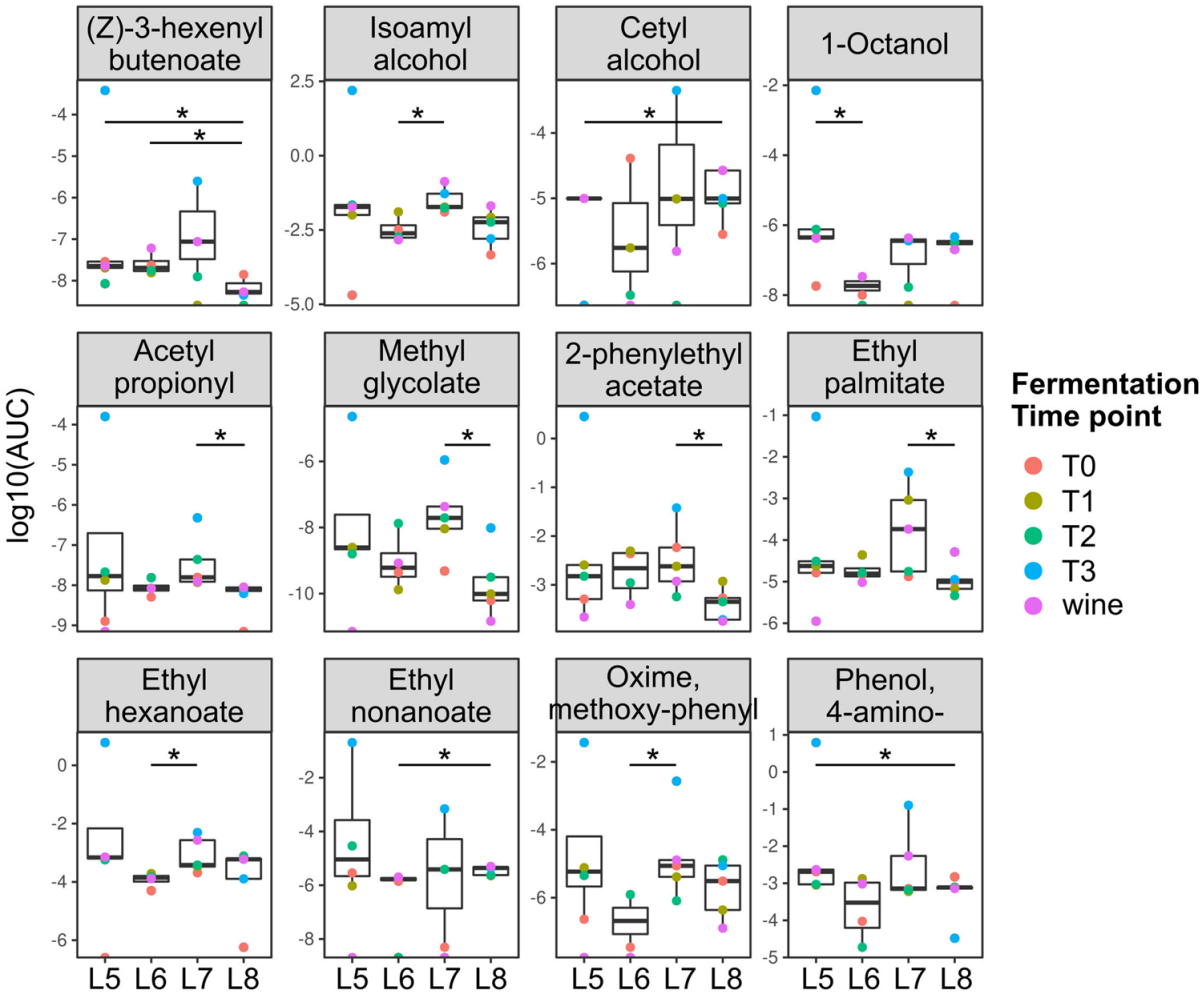
Volatile compounds significantly differing among fermentations grouped according to the vineyard row originating the grapes. Color legend indicates samples collected at different time points during fermentation. *Wilcoxon–Mann–Whitney p-value < 0.05.

Finally, we evaluated the impact of microbial changes driven by the vector-wasps on grape musts of the four experimental plant-rows by characterizing the volatilome of spontaneous fermentations (Supplementary Materials and Methods) through Gas Chromatography-Mass Spectrometry (GC–MS). Residual sugar content measured daily from start to end of the fermentation in the four batches of musts (Supplementary Fig. 5), and other parameters commonly monitored during the fermentation process (Supplementary Table 3) did not show significant differences among grape musts of the four plant-rows. The comprehensive quantification of volatile compounds present in the analyzed musts, besides highlighting the evolution of some chemicals during the alcoholic fermentation process, allowed the identification of differences associated with the pre-harvest conditions in the vineyard and potentially influencing the wine organoleptic characteristics. L8 ferments showed lower amounts of acetyl propionyl (buttery and nutty flavors), methyl glycolate, 2-phenylethyl acetate (sweet, honey, floral rosy), and ethyl palmitate (mild waxy fruity creamy) compared to L7 ferments, lower amount of (Z)-3-hexenyl butanoate (green flavor) compared to L5 and L6, and 4-amino-phenol compared to L5, but higher amounts of Cetyl alcohol compared to L5 and of ethyl-nonanoate (fruity rose) compared to L6 (Fig. 4 and Supplementary Table 4).

**Figure 5:**
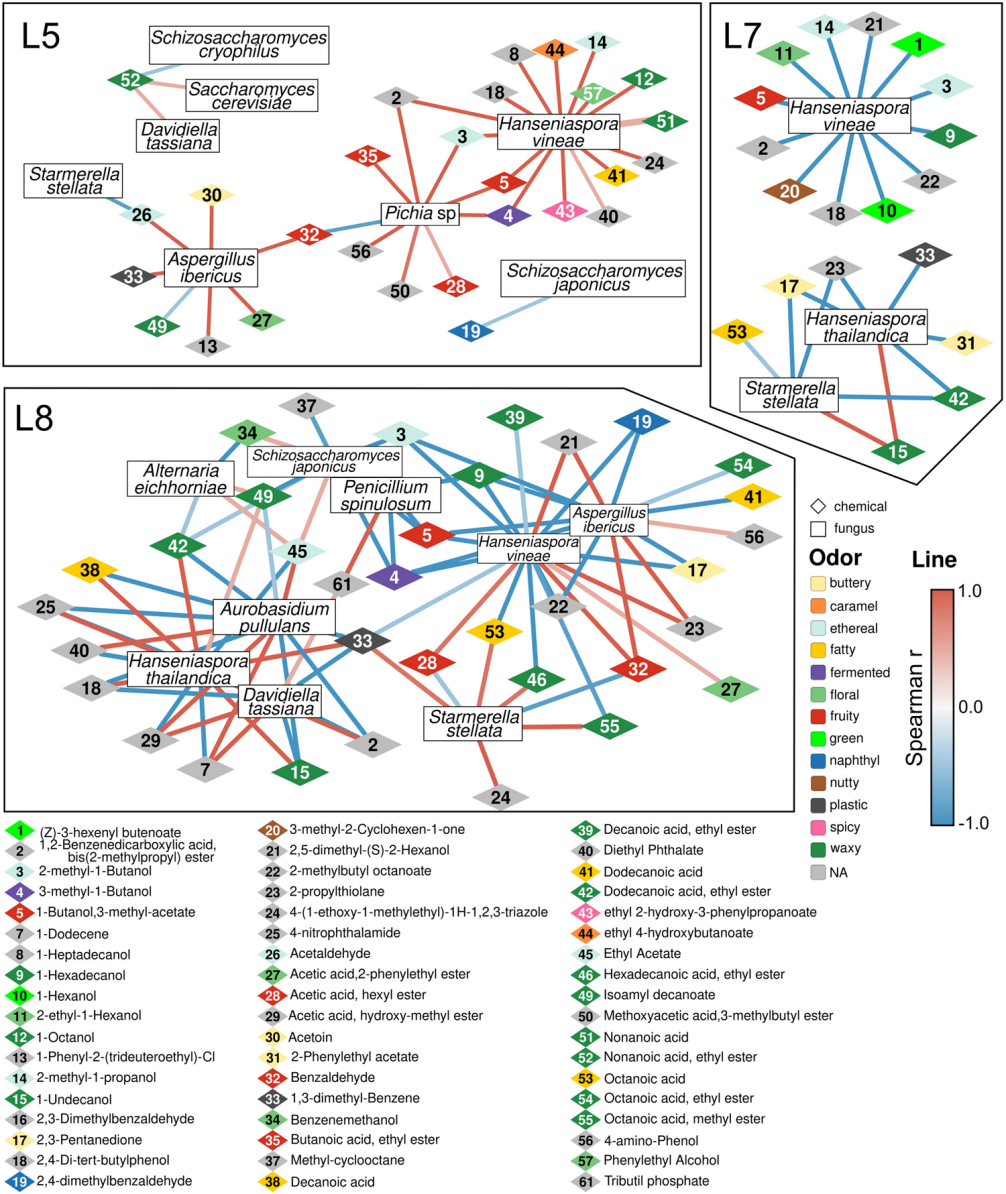
Correlations among fungal and volatile compounds abundances in fermenting musts from the studied vineyard rows. Significant Spearman correlations (r > 0.5, p-value < 0.05) with the most abundant fungi are shown, the complete list of correlations is reported in Supplementary Figs. 6–8 and Supplementary Table 5.

Conversely, the levels of isoamyl alcohol (1-Butanol, 3-methyl-; fuel oil alcoholic whiskey, fruity banana), ethyl hexanoate (sweet fruity pineapple, banana), and methoxy-phenyl oxime were higher in the L5 and L7 ferments compared to L6 and L8 ferments, whereas 1-octanol (green, citrus, floral, sweet) was more concentrated in L5 compared to L6 (Fig. 4 and Supplementary Table 4). Driven by the differences observed in the mycobiota and in the volatilomes of ferments obtained by grapes collected in the various vineyard rows, we assessed the presence of correlations between the abundance of fungi and the production of metabolites (Fig. 5). This analysis allowed us to highlight several relevant pieces of information. First of all, we could not find correlations between yeast species and volatile compounds only in the fermentation samples originating from the vineyard line covered with the net, without the inclusion of wasps (L6). This observation further supports the pivotal role of wasps in vectoring yeast strains relevant for winemaking.

The abundance of several fungi, among which *S. cerevisiae*, but also *Hanseniaspora vineae*, *Aspergillus ibericus*, and *Davidiella tassiana* in musts obtained from L5, exposed to wasps fed with the 1014 strain, was positively correlated with volatile compounds conferring floral or fruity aromas to the wine (Supplementary Fig. 6). Conversely, *H. vineae* and *H. thailandica* were negatively correlated with chemical compounds characterized by these odors in L7 ferments, exposed to the environment as the vineyard line was not covered by the net (Supplementary Fig. 7, Supplementary Table 5). These opposite correlations suggest that the inoculation of a selected *S. cerevisiae* strain through the wasp vector, not only provides per se volatile compounds oenologically relevant but can also promote the balance and contribution of natural yeasts naturally reaching the grapes (L7). The role of wasps in vectoring oenologically relevant yeasts to the vineyard grapes is further supported by the abundance of multiple species in ferments from L8 (*Alternaria eichhorniae*, *Schizosaccharomyces japonicus*, *Penicillium spinulosum*, *Aspergillus ibericus*, *H. vineae*, *Starmerella stellata*, *Aureobasidium pullulans*, *H. thailandica*, *D. tassiana*) were variously correlated, either positively or negatively, with compounds having a great range of aromas (Supplementary Fig. 8, Supplementary Table 5). In this vineyard line, exposed to wasps not fed with the selected yeasts, but isolated from the environment, the wasps may have contributed to the grape mycobiota resulting in characteristic organoleptic features not otherwise observed in naturally exposed grapes (L7) nor in sheltered grapes (L6).

## Discussion

It is now consolidated knowledge that grapes can undergo natural fermentation even without the inoculation of starter yeasts, thanks to the presence, mostly on damaged berries, of fermenting yeasts, especially *S. cerevisiae*^16^. Honeybees^17^, social wasps^11^, and the fruit fly *Drosophila*^18^ can easily carry yeasts onto ripe grapes. Furthermore, our previous study demonstrated that the hornet *Vespa crabro* and *Polistes* spp. paper wasps bear yeasts in their intestine over the winter, during hibernation and all year long, and it can vertically transmit yeasts to the offspring perpetuating this process^11,12^. Since *S. cerevisiae* is present in the gut of wasps, and wasps feed on ripe grapes, it is reasonable that wasps pierce the skin of grapes and transfer natural yeasts from their gut to the broken grapes, thereby maintaining the ecological diversity of grape’s microbiota and facilitating the spontaneous grape fermentation process^19^. Overall, these processes are extremely important in terms of insect ecology and conservation of genetic diversity of yeasts and have potential importance for winemaking and for biologic and biodynamic productions.

We demonstrated the dispersal of yeasts on vineyard grapes through *P. dominula* social wasp vectors. By taking advantage of the resistance markers of the inoculated yeast strain, we tracked the presence of this *S. cerevisiae* strain carried by wasps on ripe grapes collected at harvest, as well as in the guts of wasps released in the plant-rows. This finding proves that also social wasps with a less strong mandibular apparatus than *V. crabro*, such as *P. dominula*, when acting cooperatively, can break ripe berries and act as vectors of this yeast.

Great attention has been placed on the biotechnological potential of the insect microbiota as a bio-resource for applications in pharmaceutical and industrial contexts^17^. Thanks to their nature, flying insects have been proposed as natural vectors of microorganisms, carrying them by adhesion of cells to the cuticle or hosting cells in their intestines^11,20^. Here, we provided the first evidence that social wasps can be used as a biological tool to deliver selected yeast strain in the field. In an agro-technical context, this lays the foundation for multiple applications, such as the introduction of specific strains into a controlled area or the restoration of pre-existing microbial communities lost due to anthropogenic activity.

Metabarcoding analysis confirmed that *S. cerevisiae* was more abundant in wasps’ gut fed with the yeast strain and then released underneath the net than in wasps caught outside the net into the vineyard. This corroborates the compatibility and permanence of *S. cerevisiae* in the intestine of social wasps. The presence of wasps in the vineyard, despite not inducing a change in the richness of yeast species (alpha diversity), induced a change in the vineyard mycobiota composition, as observed through comparison of beta diversity indexes. However, it must be considered that these differences could be ascribed not only to the presence of wasps, but also to the isolation of the grapevine (and hence of grapes) from the environment due to the presence of the net, as in the absence of the net the grape mycobiota showed yet another profile.

The abundance of *S. cerevisiae* was not different among grapes obtained from the different experimental vine rows nor among grape musts, suggesting that the yeast inoculation through wasps did not alter the “natural” abundance of this species in the vineyard terroir.

The reduction of this fungal species, among the others, in wasps and samples exposed to reared wasps could be ascribed to the wide changes induced in the mycobiota of wasps reared in the laboratory. These changes mainly consist in an enrichment in *Candida* spp. and depletion in environmental species previously found in association with insects, such as *A. ibericus, C. diversa, H. thailandica, H. vineae, Issatchenkia terricola, Lachancea thermotolerans,* and *Starmerella stellata*^21^. However, the fact that these fungal species were abundant in reared wasps after the release in the vineyard suggests that the mycobiota of wasps is susceptible to the environment and highly dynamic. The decrease of fungal microbiota diversity in reared wasps holds the promises for broad application perspectives. Feeding wasps with selected fungal strains, such as strains of oenological or environmental interest, over a longer period could lead to modeling of the insect’s fungal microbiota according to the desired applicative interests. Further our results indicate that this process can be instrumental to improving the quality of the wine through the introduction of yeast strains releasing desirable aromatic profiles.

Wasps fed with TFLR *S. cerevisiae* 1014 besides being associated with changes in the composition of the vineyard mycobiota, were also associated with significant changes in the aromatic characteristics of the experimental wines. A number of these volatile compounds are very likely associated to the presence of the TFLR marker in the strain 1014. In previous publications, it has been shown that TFL mutations lead to overproduction of leucine^22–24^. Overproduction of leucine in TFLR strains has thus been shown to lead to an increase of ethyl acetate, isobutyric acid, butanoic acid, isovaleric acid, and other volatile compounds^25^. These yeast-produced aromatic compounds have also previously been shown to be attractive for insects and useful for the dissemination of yeast^20^, thus potentially providing a fitness advantage, and explaining why aromatic compounds associated to leucine metabolism are enriched in the wines from L5 (wasps fed with TFLR 1014) and L7 (without nets)*.*

Other compounds could be associated to the combined effect of the 1014 strain and other yeasts, in particular: 1-octanol (green, citrus, floral, sweet flavors) was more abundant in musts exposed to fed wasps than in musts not exposed to wasps, (Z)-3-hexenyl butanoate (green flavor) and 4-amino-phenol were more abundant compared to musts exposed to wasps not fed with *S. cerevisiae*. We also observed opposite trends in the correlations between the abundances of some fungal taxa and certain pleasant volatile compounds depending on the exposure of grapes to wasps fed with *S. cerevisiae*. In particular, the amounts of some metabolites conferring floral and fruity aromas to wines were positively correlated with the abundance of *H. vineae*, *A. ibericus*, *D. tassiana*, and *S. cerevisiae* in musts obtained from grapes exposed to wasps fed with yeast, and negatively correlated with grapes not covered with the net. Similarly, the musts obtained from grapes exposed to wasps not fed with *S. cerevisiae* showed negative correlations between *H. vineae* and several aromatic compounds conferring fatty, waxy, fruity, fermented flavors to the wine.

In summary, not only the presence of wasps is associated with a change in the organoleptic characteristics of the final product, but also the inoculation of *S. cerevisiae* through wasp-vectors coincides with significant changes in the amounts of pleasant aromas, potentially ascribed to changes in the metabolic potentials of the mycobiota.

Overall, in the ecological context, we already know how beneficial insects are important providers of biological control in vineyards allowing to create a more balanced and healthy environment preventing infestations of parasites^26^. Our results demonstrate that the yeast–insect association goes beyond the simple link between vectors and transported microorganisms. With these conclusions, it seems clear that the fact that we are living in a period in which insect populations are lower than ever before (due to pollution and the massive use of pesticides) can’t be ignored, and that the term “terroir” adopted to refer to the interaction between plants, environment, and human factors^7^ can’t leave out social insects, given their important role in the grape ecosystem and its connection with wineries.

## Methods

### Insect collection and rearing

In spring 2019, paper wasps (*Polistes dominula*) were collected using butterfly nets in the Ca’ Marcanda vineyard (43.194° Lat, 10.618° Long, Santa Teresa, Castagneto Carducci, Livorno, Italy) and then individually transferred into sterile tubes to be used for the experimental setup. For the main experiment, 12 large nests were collected at the peak of the season (mid July), deprived of the adults, and mounted in transparent sterile boxes (10 × 10 × 10 cm) with immature brood to obtain male and female callows from capped cells. Newly emerged wasps (N = 700) were fed with sterile water and sugar under controlled conditions (natural photoperiod and room temperature) until the start of the experiment. The nests were not present under the net, neither the wasps founded new nests since the experiment terminated at the harvest, in the first day of September, and the nests are produced only later in the next spring.

### Test of wasps’ ability to break the grape skin

To assess the individual wasps’ ability to pierce grape skin by single wasps, 8 wasps (4 females and 4 males) were starved for 7 days and were individually housed in a box (10 × 10 × 10 cm), with one ripe grape as the sole source of food available. To assess the cooperative ability to pierce grape skin, 8 wasp groups (4 groups of females and 4 groups of males) were housed together to feed on a single ripe grape. The time needed by the wasps alone or in groups to break the grape skin was monitored over 48 h (Supplementary Fig. 1).

#### Yeast strain used in the study as a tracer

The yeast strain used in this study was a derivative of the original *Saccharomyces cerevisiae* 1014 Castelli strain that was originally isolated from wine fermentations by Castelli in Montalcino (Siena, Tuscany, Italy) and further controlled and stabilized by Professor Mario Polsinelli and his laboratory^27,28^. The strain was selected for a dominant mutation in the Leu4 gene^29^ that made it resistant to 5,5,5-trifluoro-dl-leucine (5,5,5-TFL) and a *URA3*- auxotrophy that made it resistant to 5-Fluoro-orotic acid (5-FOA)^30^. Both resistances were derived by spontaneous mutations induced in this study and the strain has not to be considered as a genetically modified microorganism and the sequence of the strain was obtained in our previous study^31^.

The resistance markers were used to track the presence of this strain on ripe grapes, in the gut of released and recaptured wasps fed with yeast, and in musts. To this aim, the samples were plated on 5-FOA medium (0.67% Yeast Nitrogen Base with ammonium sulfate and without amino acids, 0.1% 5-Fluoro-orotic acid, 10 mM Uracil, 2% Glucose, 4% agar) and the growth of colonies assessed three days later. 5-FOA resistance colonies were then checked for 5,5,5 TFL resistance on minimal synthetic medium lacking leucine and threonine and supplemented with 200 µg/ml of 5,5,5 TFL, as previously described^28^. Since the spontaneous mutation rate to TFLR and URA- is respectively 10^–7^ and 2 × 10^–7^ the probability that a double mutant would arise during the selection procedure is so rare as to be negligible.

### Feeding wasps with *Saccharomyces cerevisiae* cells

A culture of the *S. cerevisiae* 1014 strain was prepared in YPD (1% Yeast Extract, 2% Peptone, 2% Glucose). Yeast cells were fed to a group of callow wasps (N = 320) taken from the 12 nests reared in the lab. The *S. cerevisiae* strain was provided initially ad libitum, by inoculating a sugar lump with 1014 strain cells (the inoculum was 10^8^ CFU/ml concentrated and 500 µl of it was used for each sugar lump). Right before the transfer of the wasps to the experimental field (Ca’ Marcanda vineyard), they were also individually fed with a yeast cell suspension in sucrose (10 µl of the same inoculum 10^8^ CFU/ml). Control wasps from the same nests (N = 350) were fed with sugar and sterile water only.

### Vineyard lines treatment

In the Ca’Marcanda vineyard, four adjacent rows of the grapevine of Syrah cultivar were dedicated to the field experiment and sampling. Two months before grape harvesting, three out of four grapevine rows were individually covered with a white net (0.6 mm mesh width), to prevent the entry and exit of wasps without sheltering the natural light. Two weeks later, wasp groups were released underneath the nets in two dedicated grapevine rows (L5 and L8). The experimental design was reported in Fig. 1.

### Monitoring of the microclimate, phenology, and stress of the grapevine

The potential environmental variations induced by the positioning of the net on the vine rows have been evaluated by placing a screened weather station (WS) (Data logger HOBO USB Pro T/RH mod. U23-001A) to record temperature (T, °C) and relative humidity (RH, %) at hourly time step inside the canopy of each experimental row until harvest (details in Supplementary Materials and Methods). On the 252th day of the year (DOY), leaf water potential (Bar) in the morning (9.30 a.m.) and midday (12.30 a.m.) was determined on leaves sampled from the initial, central and terminal part of both covered and not-covered row (3 leaves from each part of the row). The degree of ripeness of the grapes of each treatment was evaluated on DOY 252 determining the average sugar content of each sample (°Brix) by means of a digital refractometer (details in Supplementary Methods).

### Fungal DNA extraction and metabarcoding analysis

Total microbial DNA was extracted from the collected samples (Supplementary Table 1) by using the DNeasy Power Soil Pro kit (Qiagen) following the manufacturer’s instructions. The ITS2 region was amplified with a KAPA HiFi Taq polymerase (Roche) to carry out fungal metabarcoding analyses. To amplify the ITS2 region, the primers ITS3f (5′-GCATCGATGAAGAACGCAGC-3′) and ITS4r (5′-TCCTCCGCTTATTGATATGC-3′) were used^32^. Sequencing was carried out using an Illumina MiSeq instrument.

Raw sequences have been submitted to the ENA (European Nucleotide Archive) database with the project ID PRJEB49802-ERP134323 (Supplementary Table 1).

Raw reads were filtered and analyzed with the MICCA pipeline^33^. Briefly, primers and adapters were trimmed with the trim function, then filtered with the filter function according to the parameter values identified with the filterstats function. Metabarcoding analysis resulted in a total of 8,931,228 reads for ITS2 sequencing. Then, OTUs (Operational Taxonomic Units) were identified with the otu function (with the denovo_greedy option) and the taxonomic assignment was carried out by aligning sequences representative for each OTU against the Greengenes Core Set^34^, for bacterial analysis, or against the fungal ITS unite sequence database^35^. Once taxonomy was assigned to the identified OTUs, results were inspected to check for non-fungal (e.g., from other eukaryotes) sequences and the corresponding OTUs were ignored in the following analyses. After removal of non-fungal OTUs, samples with less than 5000 reads were pruned. Relative abundances were then calculated for each OTU in every sample as the number of reads associated with the corresponding OTU divided by the total reads obtained for the respective sample.

### Statistical analysis

To assess statistically significant differences in the grape predation test among single or group of wasps. The number of wasps, wasp's sex and their interactions were used to build a linear model and fitted by using the *lm* function. Type II ANOVA was performed using the *car* R package on the model formula^36^. Distribution of values was drawn with the *ggplot2* R package^37^.

For meteorological variables, Analysis of Variance (ANOVA) was applied to test if differences exist in the meteorological parameters amongst covered and uncovered rows. Each row was considered a single treatment while the average hourly T and RH from DOY 205–250 were used as replicates and compared using Turkey test to verify, hour by hour, if differences exist amongst treatments. As an example, the ANOVA test was applied to compare if significant differences (P < 0.05) exist amongst treatments in the hourly average T (or RH) acquired at noon along the period from DOY 205 to 250. To assess statistically significant differences among the abundances of fungal OTUs identified through metabarcoding in the different samples we carried out a differential abundance analysis with the DESeq2 R package^38^.

### Supplementary Information

Supplementary Information.

Supplementary Table 1.

Supplementary Table 2.

Supplementary Table 3.

Supplementary Table 4.

Supplementary Table 5.

Supplementary Table 6.

## Data Availability

All data supporting the findings of this study are provided in the Supplementary Information files. Raw sequences from Illumina MiSeq have been submitted to the ENA (European Nucleotide Archive) database with the project ID PRJEB49802-ERP134323 (see also Supplementary Table 1).

## References

[1] Bokulich NA, Thorngate JH, Richardson PM, Mills DA (2014). Microbial biogeography of wine grapes is conditioned by cultivar, vintage, and climate. Proc. Natl. Acad. Sci. U.S.A..

[2] Gilbert JA, van der Lelie D, Zarraonaindia I (2014). Microbial terroir for wine grapes. Proc. Natl. Acad. Sci. U.S.A..

[3] Taylor MW, Tsai P, Anfang N, Ross HA, Goddard MR (2014). Pyrosequencing reveals regional differences in fruit-associated fungal communities. Environ. Microbiol..

[4] Belda I, Zarraonaindia I, Perisin M, Palacios A, Acedo A (2017). From vineyard soil to wine fermentation: Microbiome approximations to explain the "terroir" concept. Front. Microbiol..

[5] Morrison-Whittle P, Goddard MR (2018). From vineyard to winery: A source map of microbial diversity driving wine fermentation. Environ. Microbiol..

[6] Van Leeuwen C, Seguin G (2006). The concept of terroir in viticulture. J. Wine Res..

[7] Gladstones, J. S. Introduction and definition of terroir. In *Wine, Terroir and Climate Change* (ed. Gladstones, J. S.) 1–4 (Hyde Park Press, 2011).

[8] Knight S, Klaere S, Fedrizzi B, Goddard MR (2015). Regional microbial signatures positively correlate with differential wine phenotypes: Evidence for a microbial aspect to terroir. Sci. Rep..

[9] Griggs RG, Steenwerth KL, Mills DA, Cantu D, Bokulich NA (2021). Sources and assembly of microbial communities in vineyards as a functional component of winegrowing. Front. Microbiol..

[10] Miura T, Sanchez R, Castaneda LE, Godoy K, Barbosa O (2017). Is microbial terroir related to geographic distance between vineyards?. Environ. Microbiol. Rep..

[11] Stefanini I (2012). Role of social wasps in *Saccharomyces cerevisiae* ecology and evolution. Proc. Natl. Acad. Sci. U. S. A..

[12] Stefanini I (2016). Social wasps are a Saccharomyces mating nest. Proc. Natl. Acad. Sci. U. S. A..

[13] Dapporto L (2016). Social wasp intestines host the local phenotypic variability of *Saccharomyces cerevisiae* strains. Yeast.

[14] Stefanini I, Cavalieri D (2018). Metagenomic approaches to investigate the contribution of the vineyard environment to the quality of wine fermentation: Potentials and difficulties. Front. Microbiol..

[15] Maturano YP (2015). Yeast population dynamics during prefermentative cold soak of Cabernet Sauvignon and Malbec wines. Int. J. Food Microbiol..

[16] Polsinelli M, Romano P, Suzzi G, Mortimer R (1996). Multiple strains of *Saccharomyces cerevisiae* on a single grape vine. Lett. Appl. Microbiol..

[17] Goddard MR, Anfang N, Tang R, Gardner RC, Jun C (2010). A distinct population of *Saccharomyces cerevisiae* in New Zealand: Evidence for local dispersal by insects and human-aided global dispersal in oak barrels. Environ. Microbiol..

[18] Lam SS, Howell KS (2015). Drosophila-associated yeast species in vineyard ecosystems. FEMS Microbiol. Lett..

[19] Jang S, Kikuchi Y (2020). Impact of the insect gut microbiota on ecology, evolution, and industry. Curr. Opin. Insect Sci..

[20] Christiaens JF (2014). The fungal aroma gene ATF1 promotes dispersal of yeast cells through insect vectors. Cell Rep..

[21] Meriggi N, Di Paola M, Cavalieri D, Stefanini I (2020). *Saccharomyces cerevisiae*—Insects association: Impacts, biogeography, and extent. Front. Microbiol..

[22] Cavalieri D (1998). Genetic and molecular diversity in *Saccharomyces cerevisiae* natural populations. Food Technol. Biotechnol..

[23] Casalone E, Fia G, Barberio C, Cavalieri D, Turbanti L, Polsinelli M (1997). Genetic and biochemical characterization of *Saccharomyces cerevisiae* mutants resistant to trifluoroleucine. Res. Microbiol..

[24] Brown KM (2008). Cascading transcriptional effects of a naturally occurring frameshift mutation in *Saccharomyces cerevisiae*. Mol. Ecol..

[25] Khomenko I (2017). Non-invasive real time monitoring of yeast volatilome by PTR-ToF-MS. Metabolomics.

[26] Prezoto F, Maciel TT, Detoni M, Mayorquin AZ, Barbosa BC (2019). Pest control potential of social wasps in small farms and urban gardens. Insects.

[27] Bendoni B, Cavalieri D, Casalone E, Polsinelli M, Barberio C (1999). Trifluoroleucine resistance as a dominant molecular marker in transformation of strains of *Saccharomyces cerevisiae* isolated from wine. FEMS Microbiol. Lett..

[28] Sebastiani F, Barberio C, Casalone E, Cavalieri D, Polsinelli M (2002). Crosses between *Saccharomyces cerevisiae* and *Saccharomyces bayanus* generate fertile hybrids. Res. Microbiol..

[29] Cavalieri D (1999). Trifluoroleucine resistance and regulation of alpha-isopropyl malate synthase in *Saccharomyces cerevisiae*. Mol. Gen. Genet. MGG.

[30] Boeke JD, LaCroute F, Fink GR (1984). A positive selection for mutants lacking orotidine-5'-phosphate decarboxylase activity in yeast: 5-fluoro-orotic acid resistance. Mol. Gen. Genet. MGG..

[31] Ramazzotti M (2018). Population genomics reveals evolution and variation of *Saccharomyces cerevisiae* in the human and insects gut. Environ. Microbiol..

[32] White JR, Maddox C, White O, Angiuoli SV, Fricke WF (2013). CloVR-ITS: Automated internal transcribed spacer amplicon sequence analysis pipeline for the characterization of fungal microbiota. Microbiome.

[33] Albanese D, Fontana P, De Filippo C, Cavalieri D, Donati C (2015). MICCA: A complete and accurate software for taxonomic profiling of metagenomic data. Sci. Rep..

[34] DeSantis TZ (2006). Greengenes, a chimera-checked 16S rRNA gene database and workbench compatible with ARB. Appl. Environ. Microbiol..

[35] Abarenkov K (2010). The UNITE database for molecular identification of fungi—Recent updates and future perspectives. New Phytol..

[36] Fox, J., Weisberg, S. & Fox, J. *An R Companion to Applied Regression*, 2nd ed (SAGE Publications, 2011).

[37] Wickham, H. *Ggplot2: Elegant Graphics for Data Analysis*. (Springer, 2009).

[38] Love MI, Huber W, Anders S (2014). Moderated estimation of fold change and dispersion for RNA-seq data with DESeq2. Genome Biol..

