# Supplementary material for: Using wasps as a tool to restore a functioning vine grape mycobiota and preserve the mycobial “terroir”
Source: Sci Rep. 2023 Oct 2;13:16544. doi: 10.1038/s41598-023-43541-9 (PMC10545793; doi:10.1038/s41598-023-43541-9)
Supplement: Supplementary file 1 — Supplementary Information. [file 41598_2023_43541_MOESM1_ESM.pdf]

Supplementary information for

Using wasps as a tool to restore a functioning vine grape mycobiota and preserve the mycobial "terroir"

Monica Di Paola[1$], Agnese Gori[1 $], Irene Stefanini[2], Niccolò Meriggi[1], Sonia Renzi[1], Stefano Nenciarini[1], Benedetta Cerasuolo[1], Marco Moriondo[3], Riccardo Romoli[4], Giuseppe Pieraccini[4], David Baracchi[1], Francesco Turillazzi[5], Stefano Turillazzi[1,5], Duccio Cavalieri[1,5*]

Affiliations

[1] Dept. of Biology, University of Florence, via Madonna del Piano 6, Sesto Fiorentino, 50019, Florence, Italy
[2] Dept. of Life Sciences and Systems Biology, University of Turin, Turin, Italy
[3] National Research Council, Bioeconomy Institute, Sesto Fiorentino, 50019, Florence, Italy
[4] Mass Spectrometry Centre (CISM), University of Florence, via U. Schiff, 6, Sesto Fiorentino, 50019, Florence, Italy
[5] LABREMMA-Laboratory for medical entomotherapy, microbiology and environment, University of Florence, Sesto Fiorentino, 50019, Florence, Italy

$ Equal contribution

* Corresponding author:
Duccio Cavalieri
University of Florence Dept. of Biology
Email: duccio.cavalieri@unifi.it

This pdf includes:

Supplementary Text

Supplementary Figure 1

Supplementary Figure 2

Supplementary Figure 3

Supplementary Figure 4

Supplementary Figure 5

Supplementary Figure 6

Supplementary Figure 7

Supplementary Figure 8

Supplementary Text

Monitoring of microclimate and plant stress of plant-rows covered and not covered by nets

The results on temperature (T; °C) and relative humidity (RH; %) monitoring highlighted significant differences in general between the reference weather station (WS) and those placed in the canopy of rows starting from 9AM to 4PM, irrespective of the treatment. This result may be addressed to the different distance from the ground at which the WSs were placed. In general, the reference WS, placed at 2 meter height, recorded lower temperatures as compared to those placed in the canopy that were closer to the soil and therefore more affected by the heating of this during the central part of the day. Differences in T and RH were occasionally found during the day between covered rows, as the likely effect of local conditions were the WSs were placed and considering that the vineyard rows were North-South oriented implying that the sun's rays strike orthogonally on the row, on the side facing East, until noon and on the West side, in the afternoon. This could create anomalous behaviors in the case in which the boundary conditions of every row differ during the day. As an example, although L7 (plant- line covered by net) exhibited a daily T trend overlapping with other rows up to 3PM, it has significantly lower temperature values than the other plant-lines in the afternoon part of the day as the effect of a higher shading of the immediately adjacent row with respect to the other rows (**Supplementary Figure 2**). However, despite these local conditions may play an important role in determining the variation in T and RH, the uncovered row does not show significant differences in any part of the day compared to any of the covered ones, indicating that the treatment did not affect the values recorded inside the canopy.

The same applied to fraction of radiation intercepted by the canopy (Fapar), where L7 (net-free) and L5 (covered by net) exhibited a decreasing leaf area interception from veraison (0.15±0.052 and 0.157±0.052, respectively) to harvest (0.10±0.06 and 0.11±0.02) with no significant differences between treatments.

Comparison of microbiota diversity among terroir, grapes and wasps

Differences among the mycobiota of samples collected in the studied vineyard rows were evaluated (**Supplementary Table 2E** and Supplementary Information). Whereas any of the identified fungal species were found specifically associated with one of the groups of samples (either grouped according to the vineyard row or to the type of specimen), *Davidiella tassiana*, an endophytic species commonly found associated with *Vitis vinifera* (Pancher et al. 2012), was the only species found in every analyzed sample. In addition, *Hanseniaspora uvarum* was present in every must sample, *H. thailandica* in every grape sample, and *Aureobasidium pullulans, Saccharomyces cerevisiae* and *Yarrowia lipolytica* were present in every laboratory wasp. Some fungal taxa were consistently present in samples originating from the same vineyard row, such as *A. pullulans* was present in every L5 sample, *H. thailandica*, *H. occidentalis*, *Alternaria eichhorniae*, and *H. vineae* (also present in every L7

sample) were present in every L6 samples. *A. eichhorniae* was also present in every L8 sample (**Supplementary Table 3**).

Supplementary Materials and Methods

Monitoring of the microclimate, phenology, and stress of the grapevine

The potential environmental variations induced by the positioning of the net on the vine rows have been evaluated by placing a screened weather station (WS) (Data logger HOBO USB Pro T/RH mod. U23-001A) to record temperature (T, °C) and relative humidity (RH, %) at hourly time step inside the canopy of each experimental row until harvest. The WSs were placed in the central part of each row at a distance from the ground of about 0.7 m, taking care to select homogeneous areas in terms of leaf area index (LAI). A reference weather station was placed above the canopy (2-meter height) for standard temperature and humidity measurements (**Supplementary Fig. S2**). The effect of shadowing on leaf area development was monitored in row 7 (net-free) and 5 (covered by net) on day of year (DOY) 237 and 244 by evaluating the fraction of radiation intercepted by the canopy (Fapar) sampled using a ceptometer (Accupar, LP80). This measured incoming and below canopy radiation at 1 meter intervals along the rows (20 measurements for each row). On DOY 252, leaf water potential (Bar) in the morning (9.30 AM) and midday (12.30 AM) was determined on leaves sampled from the initial, central and terminal part of both covered and not-covered row (3 leaves from each part of the row). The degree of ripeness of the grapes of each treatment was evaluated on DOY 252 by composing the bunches from the initial, central and terminal part of each row and determining the average sugar content of each sample (° Brix) by means of a digital refractometer.

Grape fermentation

After the harvest, 90 Kg of ripe grapes were collected in sterile tanks from each experimental grapevine row separately to avoid cross-contamination and then mechanically pressed. Grape musts were left in inox drums (100 liters capacity) to ferment separately and spontaneously (with the only addition of 2% tartaric acid). Spontaneous fermentation was monitored daily by measuring the residual sugar content (g/L) as an indirect measure of ethanol production. Acetic, tartaric and malic acids production (g/L) and pH were also measured. During fermentation, samples at multiple time points were collected until the end of alcoholic fermentation (when the must sugar content was equal to 0) for metabarcoding analysis.

Supplementary Figures

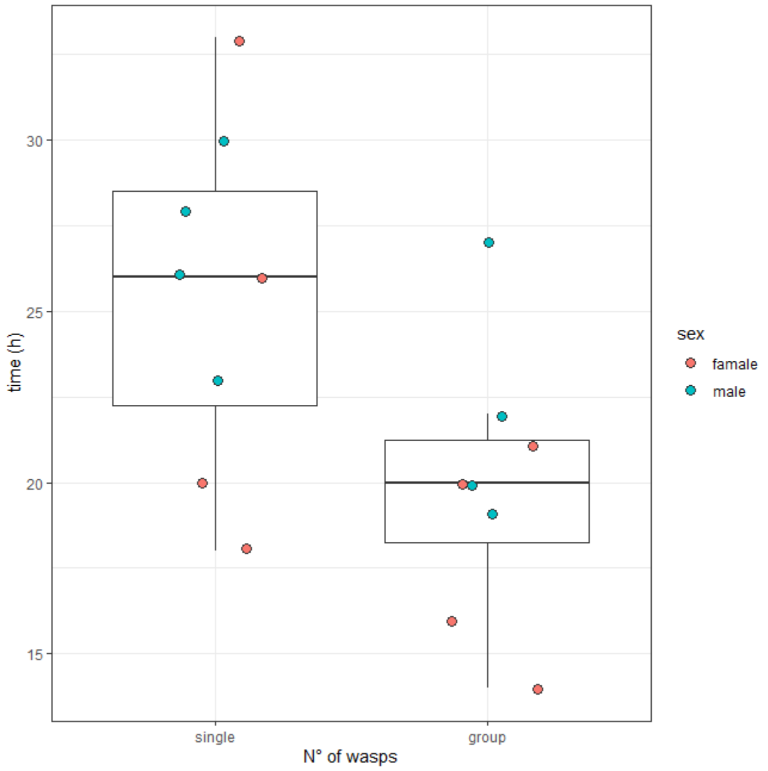

Supplementary Figure 1. Grape predation test. Boxplot of the time spent from single wasps or groups of wasps to bite the grape berries. Time (h) was reported on y-axis, while sample groups were reported on x-axis. Wasp's sex was reported in the legend following color pattern. Grape berries were the unique food source in the box. [N° of wasps, ANOVA test p=0.026].

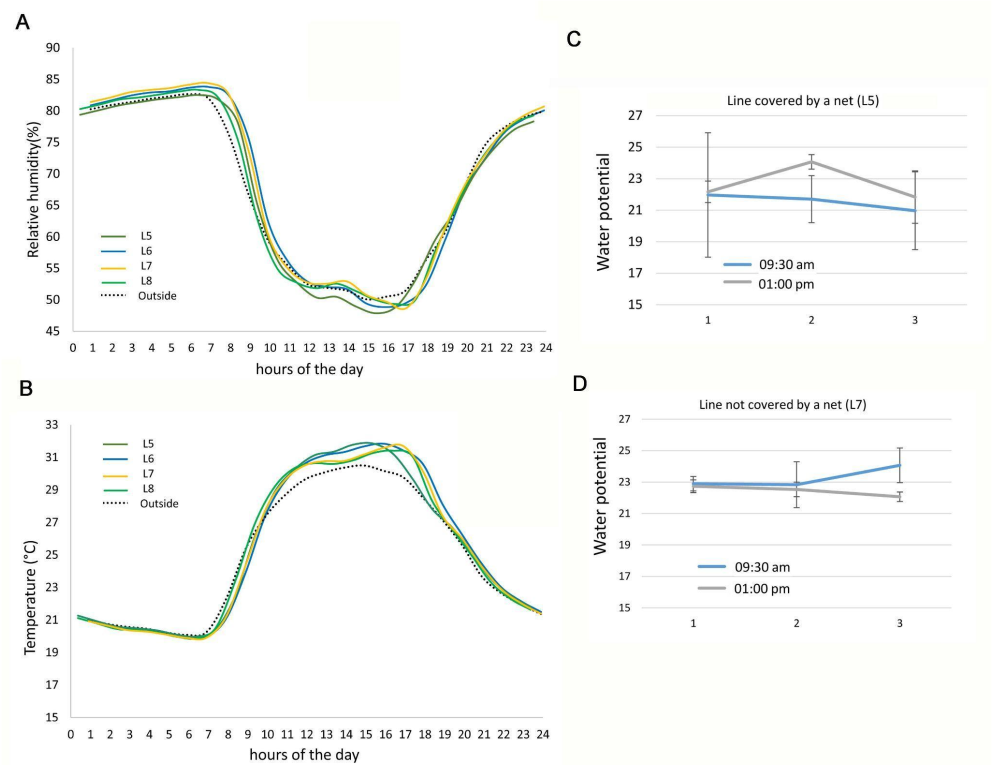

Supplementary Figure 2. Monitoring of climate and plant stress. Evaluation of (A) relative humidity and (B) temperature measured into the four plant- rows and outside from the experimental vine rows. For each hour of the day, the mean of values measured over the 7 weeks of experiment are reported. (C-D) Water potential of vine plants tested (C) in a vine row covered by a net (L5), and (D) in a vine row not-covered by net (L7). Measurements were performed at 09:30 am and 01:00 pm (mean +/- standard deviation) in three different parts of the grapevine row (at the two ends and in the middle).

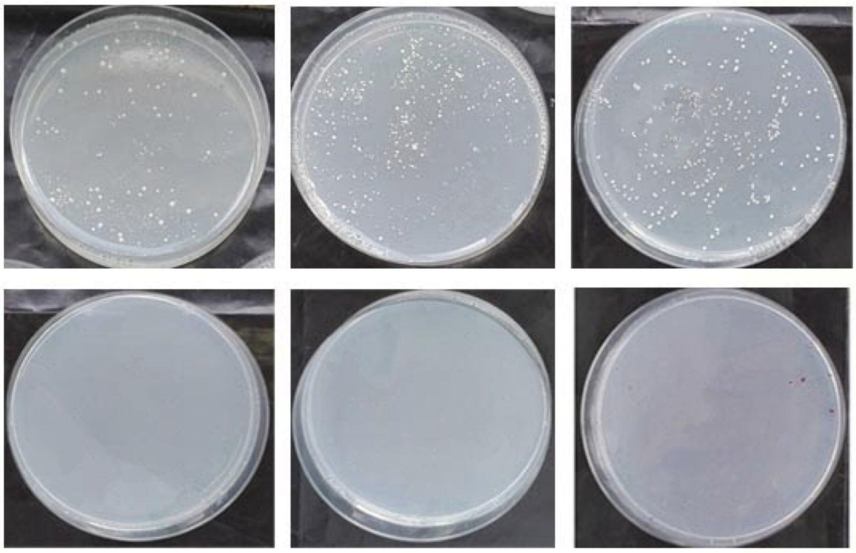

Supplementary Figure 3. Tracking of the vectored *S. cerevisiae* strain into wasps gut and grapes. In order to track the *S. cerevisiae* strain carried by wasps on gut and on grapes, we took advantage of the 5-FOA resistance of the *S. cerevisiae* 1014 strain (URA3-) inoculated into the wasps-vectors. We plated grapes individually collected from each experimental plant row onto YNB medium with 5-FOA, and we checked the growth of the resistant colonies of yeast strain. The figure shows as an example, 5-FOA resistant colonies of *S. cerevisiae* 1014 strain isolated into the gut of wasps released into the two experimental vine rows and caught at harvest. On the top, yeast colonies isolated from intestines of wasps fed with yeast (caught underneath the net of L5 plant row). On the bottom panel, yeast colonies isolated from intestines of wasps not fed with yeast (caught underneath the net of L8 plant row). Resistant colonies were observed after 3 days. The picture shows colony growth from 6 wasp guts. One wasp gut sample in each plate.

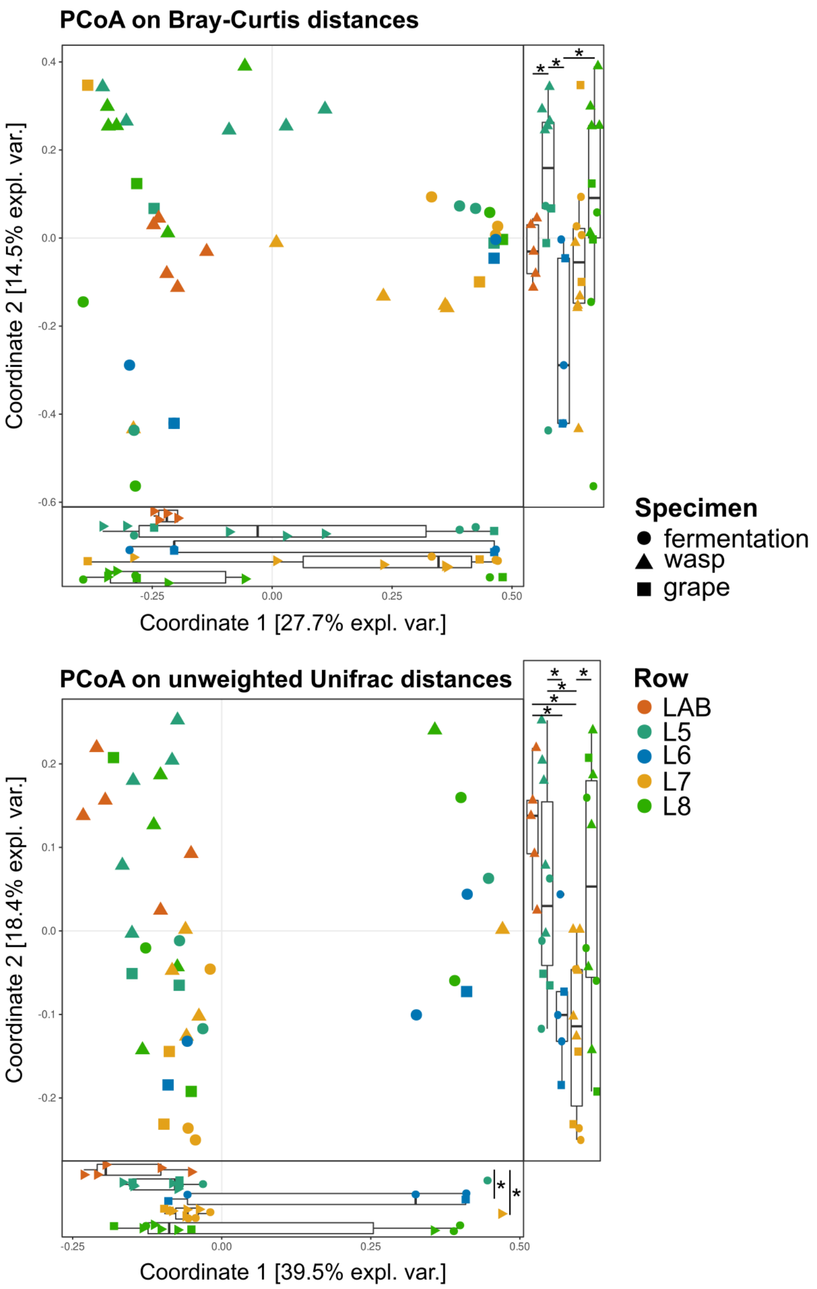

Supplementary Figure 4. Beta diversities - PCoA on Bray-Curtis (upper) and unweighted UniFrac (lower) distances. *=Wilcoxon-Mann-Whitney p-value<0.05

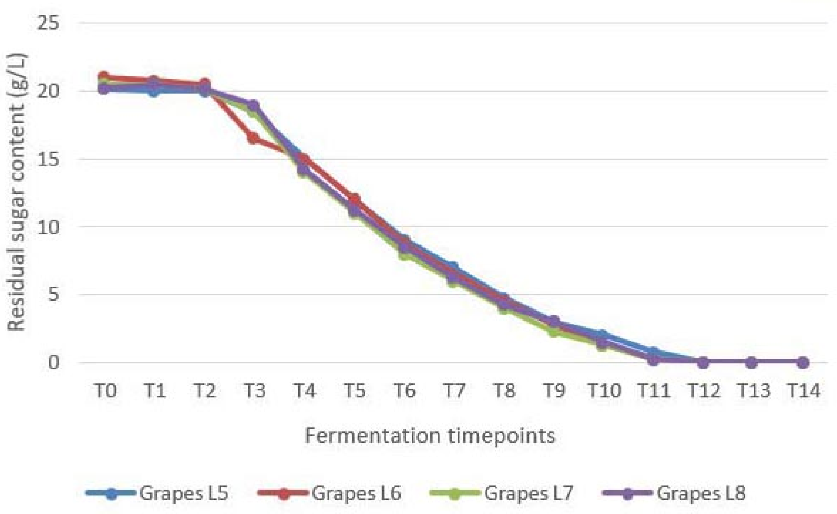

Supplementary Figure 5. Grapes fermentation. Comparison of the residual sugar content during the fermentation process of musts from grapes of the four vine rows.

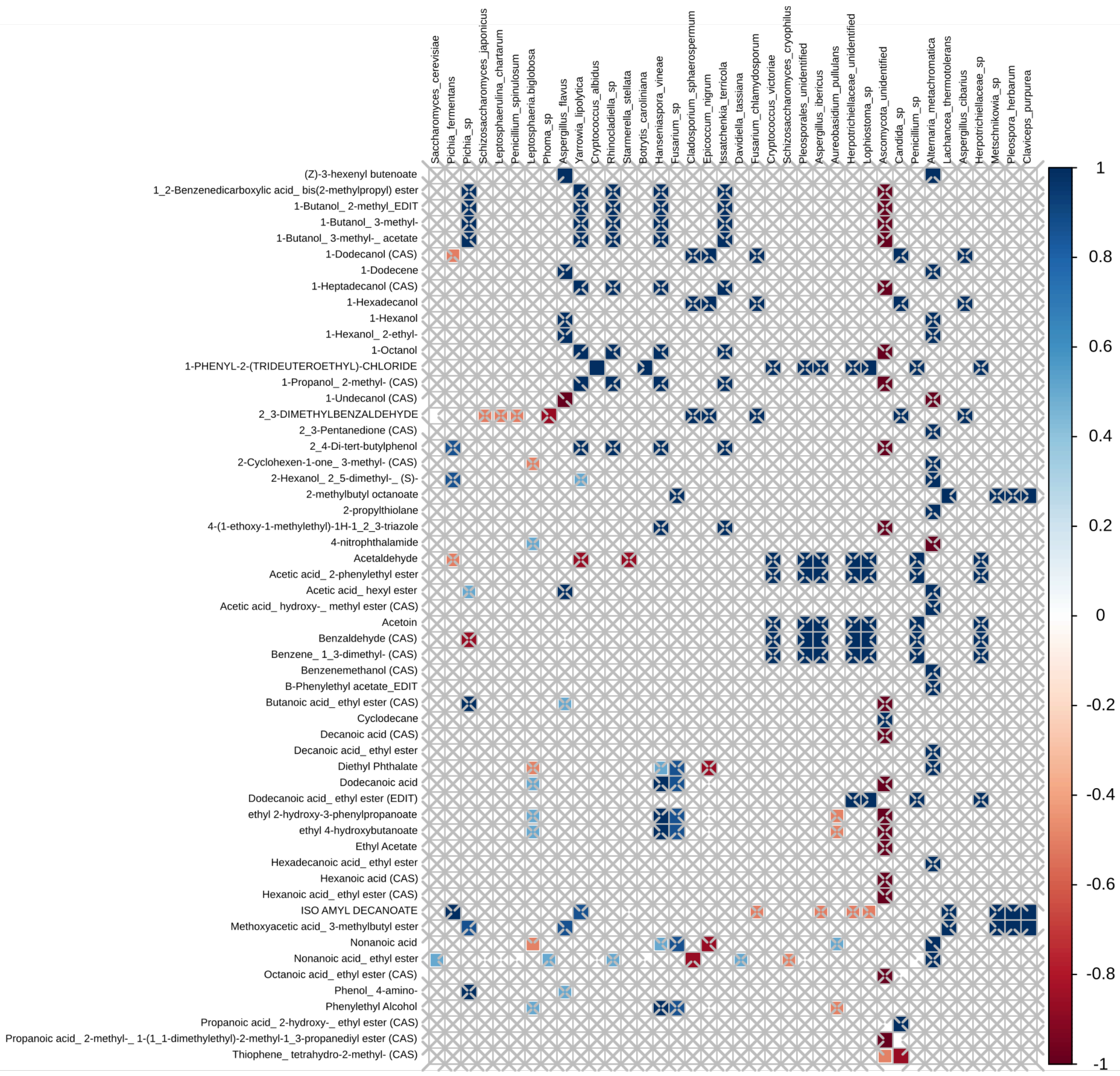

Supplementary Figure 6. Correlations among fungal and volatile compounds abundances in fermenting musts from vineyard line L5. Significant Spearman correlations (r>0.5, p-value<0.05) are shown. Crossed squares indicate not significant correlations.

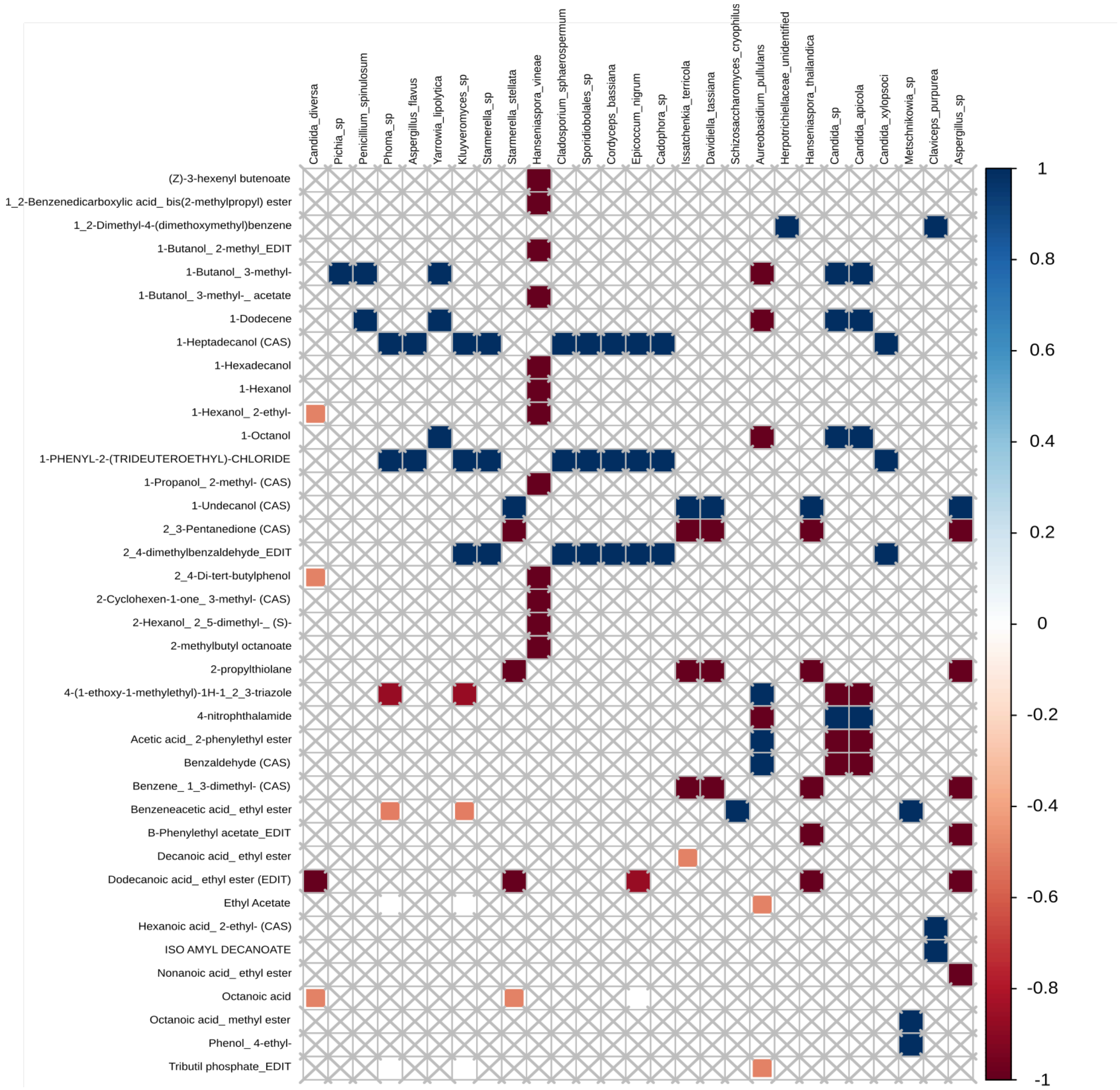

Supplementary Figure 7. Correlations among fungal and volatile compounds abundances in fermenting musts from vineyard line L7. Significant Spearman correlations (r>0.5, p-value<0.05) are shown. Crossed squares indicate not significant correlations.

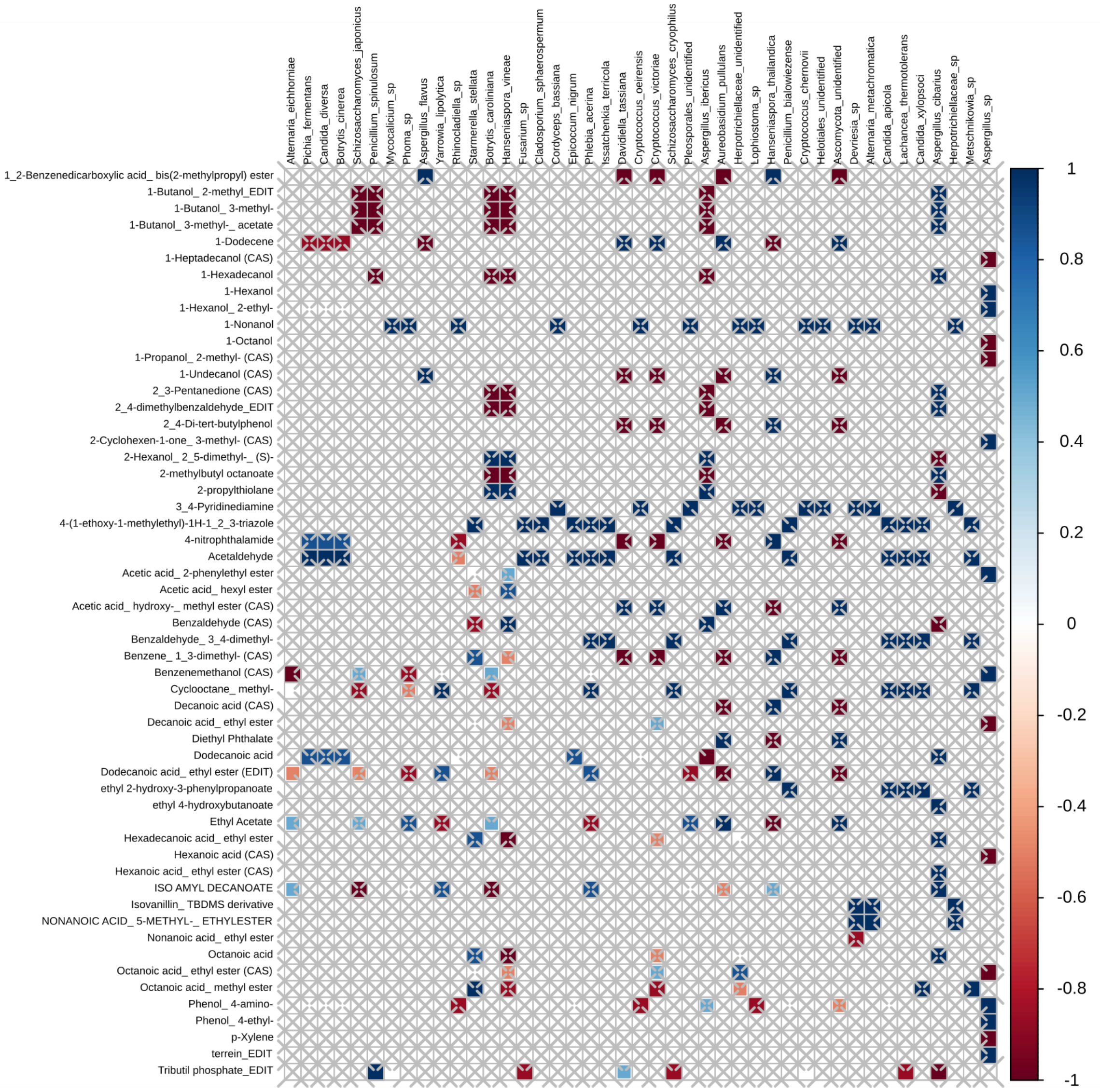

Supplementary Figure 8. Correlations among fungal and volatile compounds abundances in fermenting musts from vineyard line L8. Significant Spearman correlations (r>0.5, p-value<0.05) are shown. Crossed squares indicate not significant correlations.
